# Polydopamine-Mediated Protein Adsorption Alters the Epigenetic Status and Differentiation of Primary Human Adipose-Derived Stem Cells (hASCs)

**DOI:** 10.3389/fbioe.2022.934179

**Published:** 2022-08-10

**Authors:** Javad Harati, Xuelian Tao, Hosein Shahsavarani, Ping Du, Massimiliano Galluzzi, Kun Liu, Zhen Zhang, Peter Shaw, Mohammad Ali Shokrgozar, Haobo Pan, Peng-Yuan Wang

**Affiliations:** ^1^ Shenzhen Key Laboratory of Biomimetic Materials and Cellular Immunomodulation, Shenzhen Institute of Advanced Technology, Chinese Academy of Sciences, Shenzhen, China; ^2^ Shenzhen College of Advanced Technology, University of Chinese Academy of Sciences, Beijing, China; ^3^ Lab Regenerative Medicine and Biomedical Innovations, Pasteur Institute of Iran, Tehran, Iran; ^4^ Department of Cell and Molecular Biology, Faculty of Life Science and Biotechnology, Shahid Beheshti University, Tehran, Iran; ^5^ Materials Interfaces Center, Shenzhen Institutes of Advanced Technology, Chinese Academy of Sciences, Shenzhen, China; ^6^ Oujiang Laboratory, Key Laboratory of Alzheimer’s Disease of Zhejiang Province, Institute of Aging, Wenzhou Medical University, Wenzhou, China

**Keywords:** polydopamine (PDA), adipose stem cells ASCs, integrin, differentiation, epigenetic status

## Abstract

Polydopamine (PDA) is a biocompatible cell-adhesive polymer with versatile applications in biomedical devices. Previous studies have shown that PDA coating could improve cell adhesion and differentiation of human mesenchymal stem cells (hMSCs). However, there is still a knowledge gap in the effect of PDA-mediated protein adsorption on the epigenetic status of MSCs. This work used gelatin-coated cell culture surfaces with and without PDA underlayer (Gel and PDA-Gel) to culture and differentiate primary human adipose-derived stem cells (hASCs). The properties of these two substrates were significantly different, which, in combination with a variation in extracellular matrix (ECM) protein bioactivity, regulated cell adhesion and migration. hASCs reduced focal adhesions by downregulating the expression of integrins such as αV, α1, α2, and β1 on the PDA-Gel compared to the Gel substrate. Interestingly, the ratio of H3K27me3 to H3K27me3+H3K4me3 was decreased, but this only occurred for upregulation of *AGG* and *BMP4* genes during chondrogenic differentiation. This result implies that the PDA-Gel surface positively affects the chondrogenic, but not adipogenic and osteogenic, differentiation. In conclusion, for the first time, this study demonstrates the sequential effects of PDA coating on the biophysical property of adsorbed protein and then focal adhesions and differentiation of hMSCs through epigenetic regulation. This study sheds light on PDA-mediated mechanotransduction.

## Introduction

Polydopamine (PDA) is a bioadhesive polymer that has been widely used for biomedical devices and biomaterials due to its low cost, biocompatibility, and simple coating process that requires soaking the material in an aqueous alkaline solution of dopamine (Lee et al., 2007). The impact of PDA has been thoroughly investigated previously (Zhu et al., 2019; Deng et al., 2021c, Deng et al., 2021 K.). Based on the previous evidence, PDA coating promotes mesenchymal stem cell (MSC) differentiation (Lee et al., 2014, 2017; Kong et al., 2020). These results are related to improved wettability, cell attachment, proliferation, and migration on the PDA-coated surfaces compared to an untreated surface. Nevertheless, the influence of PDA coating on extracellular matrix (ECM) protein adsorption and its property on the epigenetic state of stem cells remains unexplored.

Surface coating with PDA leads to changes in the surface properties of a material, such as the mechanical properties and structural features (Sharma et al., 2019). During PDA coating, the thickness depends on concentration and time (i.e., 25 nm for 1 mg/ml). In addition, aggregated PDA particles often appeared which altered the surface roughness. The PDA structure is a supramolecular structure generated by H-bonding and π–π interactions between dopamine monomers, with trimers and tetramers being the most common kinds (Liebscher et al., 2013; Ryu et al., 2018; Liebscher, 2019). The outcome of the PDA-modified cell culture substrate (e.g., roughness, mechanics, and adsorbed proteins) would alter the ultimate behavior of the stem cells, such as differentiation. For example, PDMS coated with PDA, and then collagen (Col) has been shown to facilitate osteogenic differentiation of MSCs by increasing alkaline phosphatase (ALP) expression (Chuah YJ. et al., 2015). Another study constructed an ECM-mimicking hydrogel scaffold by incorporating polydopamine-modified hyaluronic acid (PDA/HA) complex into a dual-crosslinked collagen (Col) matrix for growth factor-free cartilage regeneration (Gan et al., 2022). Indeed, PDA is rarely used as a sole material for stem cell culture and is often combined with proteins or polysaccharides to improve the overall bioactivity and differentiation efficiency. Stem cell differentiation is a long-term cellular response to the microenvironment, including surface topography, stiffness, wettability, and bioactivity, and these parameters are affected by PDA coating in a concentration-dependent manner. In addition, PDA coating can increase the stability of the absorbed proteins and biomolecules, which is accompanied by a conformational and bioactivity change in the absorbed proteins (Secundo, 2013; Ball, 2014; Harati et al., 2017).

The specific receptors first sense the mechanical property of surfaces on the cell surface (e.g., integrins). The sensors then trigger the machinery of signaling pathways [e.g., the focal adhesion (FAs) and PI3K pathway], which in turn activate or repress transcription factors that regulate cell fate (Chien, 2007; Wang et al., 2016). Therefore, cell surface receptors such as integrins have been intensively studied for stem cell differentiation. Priming of α5-integrin, for example, has been found to increase osteogenesis (Hamidouche et al., 2009), and upregulation of the PI3K pathway has been demonstrated to be involved in the downstream of integrin signaling, leading to osteogenesis promotion (Lee et al., 2014). The relationship between integrins and the epigenome helps explain how environmental inputs are translated into changes in gene expression and, ultimately, stem cell function and differentiation (Dalby et al., 2014).

This research aims to investigate the influence of the PDA on proteins that subsequently alter stem cell differentiation. PDA coating can directly change protein adsorption; as a result, cell adhesion, migration, gene expression, and the differentiation of human adipose stem cells (hASCs) can be very different. Gelatin was applied in this study as a common and cost-effective protein derived from collagen for stem cell cultivation. For the first time, we explore the mechanism of stem cell–PDA interaction from an epigenetic point of view. This study provides new insights into PDA coating and its potential application in stem cells and regenerative medicine.

## Materials and Methods

### Substrate Fabrication

All substrates were treated with air plasma (300 W) for 3 min. Tissue culture plates (TCP, Corning Inc. United States) were coated with polydopamine (dopamine hydrochloride, cat. no. D103111, Aladdin, dissolved in 10 mM Tris buffer (pH 8.5) at 1.5 mg/ml) for 1.5 h in an orbital shaker. Tris buffer was used for the control substrate during PDA coating. Substrates were washed three times with distilled water and then sterilized with 75% ethanol for at least 20 min. Gelatin (type A from porcine skin, Yeasen Biotech Co. Ltd., China) 0.1% (wt) was used to coat the surface for 30 min at 37°C.

### Atomic Force Microscopy

The local elasticity was probed with a commercial AFM JPK Nanowizard4 (Bruker, United States) in the force volume (FV) mechanical imaging mode. A force curve (FC) is obtained in an FV experiment by vertically indenting the AFM-tip at every point of a regular matrix in the selected area, which yields the local mechanical response at every point probed. The morphology is reconstructed from the FV map producing a 1:1 correspondence between the morphology and mechanical properties. A series of 4–5 FV measurements were performed in different macroscopic positions to improve the statistical reliability of the experiments.

All samples were imaged while immersed in DMEM at 37°C after waiting 20 min for thermal equilibration. A probe model SNL was used considering 20 nm of nominal contact radius for all samples. The thermal noise method was used to calibrate the elastic spring constant, k = 0.36 n/m (Butt and Jaschke, 1995). Young’s moduli were evaluated by data analysis performed with a custom software “AFMech Suite” environment with a routine fully described previously (Galluzzi et al., 2016, 2018).

For roughness measurement and probe parameters, k = 0.7 n/m and R = 10 nm were used. For each sample, five maps (at 20 μm lateral scan size) were acquired in different macroscopic positions. The root mean square roughness R_Q_ is a parameter usually used to describe ns-surfaces following the relation:

$$R_{Q}=\;\sqrt{\frac{1}{n}\sum_{i=0}^{n}{\left(x_{i}-\bar{x}\right)}^{2}}\text{ ,}$$ (1)

where n is the number of points on the map, x is the local height value, and $\bar{x}$ is the average height of the plane.

### Water Contact Angle

WCA measurements were performed using a goniometer (Biolin Scientific, Sweden). The PDA and PDA-Gel surfaces were dried under a cell culture hood for 30–60 min and then cut into a suitable size for the measurement. WCA was measured using a 3 μL distilled water droplet at room temperature, and the angles were recorded after 5 s. Three measurements per sample were averaged (*n* = 3).

### X-Ray Photoelectron Spectroscopy

X-ray photoelectron spectroscopy (XPS) was carried out on an ESCALAB 250Xi spectrometer (Thermo Fisher Scientific Inc.), which was equipped with a monochromatized Al K source with a power of 150 W (15 kV), a hemispherical analyzer in the fixed analyzer transmission mode, and a standard aperture with an analysis area of 0.5 mm × 0.5 mm. The total pressure in the main vacuum chamber was kept in the 10^–10^ m bar range throughout the study. Survey spectra were collected with a pass energy of 100 eV, while high-resolution C1s, N1s, O1s, and S2p spectra were collected with a pass energy of 20 eV. After Ar sputtering, a depth profile of 20 nm was achieved (etching area: 2.5 mm × 2.5 mm, etching rate 1 nm/s, 20 s). Thermo Advantage software was used to process the data.

### hASC Culture

Primary human adipose-derived stem cell (hASC) passages 4–6 were collected from lipoaspirates at National Taiwan University Hospital (Prof Naichen Cheng). In this study, the cells were cultured for 5 days in a growth medium (GM). GM consists of Minimal Essential ALPHA basic medium (Gibco, C11995500BT) supplemented with 10% (v/v) fetal bovine serum (FBS, Gibco, 1099141C) and 1% antibiotics (100 units/mL penicillin and 100 μg/ml streptomycin, Hyclone, SV30010). For differentiation, the medium was replaced with a differentiation medium (DM, Supplementary Table S1 **),** and the medium was replaced every 2 days.

### Immunostaining

For FA staining, cells were seeded at a cell density of 15,000 cells/cm^2^ and cultured for 24 h. The cells were fixed with 4% paraformaldehyde (PFA) for 20 min, permeated with 0.2% Triton-100 in PBS for 5–10 min, and then blocked in 1% bovine serum albumin for 1 h. Samples were incubated with the primary antibody of mouse monoclonal anti-vinculin (v9131; Sigma-Aldrich, 1:400 dilution in 1% BSA) at 4°C overnight. After three times of wash with PBS, the samples were incubated with the secondary antibody of goat anti-mouse IgG-H&L FITC for 2 h (ab6785, Abcam, 1:1,000 dilution in PBS). Cell nuclei were stained using DAPI (500 ng/ml, Dojindo, D523, company, China).

### Live Cell Imaging

Viral-transduced GFP-positive hASCs were cultured on the substrates in a 24-well plate for 24 h before it was used for live-cell imaging using a ×10 objective lens (JuLI Stage, PE, United States). ImageJ software (1.53f53) with a manual tracking option was used to analyze the videos made of 30 min interval live-cell imaging. For comparison, the average speed of each cell was calculated. Three positions were imaged per sample (*n = 3*).

### Cell Adhesion and Proliferation

Cell proliferation was measured at different time points using CCK-8 (CC0039, Beyotime Biotechnology, China) according to the manufacturer’s instructions. In brief, cells were seeded at a density of 15,000 cells/cm^2^ in 24-well plates and incubated in a cell incubator for various time points: 3, 24, 48, 72, and 96 h. At each time point, cells were incubated in a 10% CCK-8 reagent in GM for 2 h at 37°C. Then, the suspension was collected for absorbance measurement at a wavelength of 450 nm using a microplate reader (Infinite ^®^ 2000 PRO, Tecan, Switzerland). Next, the cell number was measured with the help of a standard curve. Lastly, the CCK-8 assay was performed with three replicates (*n* = 3).

### Cell Adhesive Measurement

After seeding the cells on the surfaces for 24 h, sonication was applied for 30 s, with a frequency of 1 MHz and a power of 1 W/cm^2^ (SXUltrasonic, China). To calculate the cell number, we stained the cell nucleus with Hoechst (1 μg/ml). Following this ultrasonic treatment, the medium was immediately changed, and the images of the samples were captured using the Molecular Image Xpress Micro Confocal System (10x optical lens, Molecular Devices, LLC, United States). Cell numbers around the center of the samples were counted using the particle analysis module in ImageJ software (1.53f53). For each sample, at least 20 images of three samples were analyzed (*n* = 3).

### Oil Red Staining

The differentiated cells were fixed for 30 min at room temperature with 4% PFA and then rinsed with phosphate-buffered saline. The oil Red staining solution is prepared by dissolving 0.5% oil red in propylene glycol (O1391-250ML, Sigma-Aldrich, United States) and filtering with the filter paper (protected from light), staining the lipid droplets for 30–60 min, and then washing with distilled water. The absorbance at 520 nm was measured after extracting the dye with isopropanol. Normalizing lipid signal (Oil red-O) to cell number was performed by counting the number of Hoechst-stained cell nuclei.

### Alizarin Red S Assay

The differentiated cells were fixed using 4% PFA. After thoroughly rinsing, the samples were stained for 15 min at room temperature with 1% Alizarin Red S (A5533, Sigma-Aldrich, United States) in Tris–HCL (pH = 4.2). After that, samples were thoroughly washed with deionized water three times and examined under a bright-field microscope. Additionally, the Alizarin Red S stained samples (*n* = 2) were extracted *via* 10% (w/v) cetylpyridinium chloride (PHR1226, Sigma-Aldrich, United States) in PBS, and then the absorbance of the extracted color was assessed at 590 nm.

### Alcian Blue

In brief, the differentiated cells were fixed with 4% PFA for 30 min at room temperature, and then DPBS was used to rinse the cells. 3% Alcian Blue 8GX (Sigma, A9186, United States) in acetic acid was allowed to react for 90 min at room temperature to identify GAG deposition of differentiated chondrocytes. After washing with distilled water three times, images were taken for analysis.

### GAG Assay

After 21 days of chondrogenesis, the differentiated cells were harvested and digested for 16 h at 57°C in a 1.5-ml Eppendorf tube with 1 mg/ml papain prepared in 0.1 M phosphate buffer with 10 mM Na-EDTA and 10 mM l-cysteine-HCl (pH = 6). To measure the number of sulfated GAGs, 100 µL of the final solution was mixed with a solution of 100 µL of dimethyl methylene blue (DMMB, Sigma, 341,088), and the ODs were then measured at 530 nm by a microplate reader (Multiscan, Thermo Fisher). For the DMMB solution preparation, 0.8 mg DMMB, 0.1 g sodium formate, and 0.1% formic acids were added in 50 ml PBS. To quantify the result, we used a standard curve of chondroitin 6-sulfate. For normalization, we measured the DNA amount with a PicoGreen kit (P11496, Thermo-Fisher Scientific, United States).

### qPCR

Cells were lysed in TRIzol (Invitrogen, CA, United States), and RNA was extracted according to the manufacturer’s instructions. Complementary DNA was synthesized using a cDNA synthesis kit (Takara, RR037A, Japan). The reactions were set up in triplicate with the SYBR FAST qPCR Kit (GeneStar, China) and run on a Roche PCR system (Germany). The gene expressions were normalized to the housekeeping gene, β-Actin. Primer sequences are listed in Supplementary Table S2.

### BCA Assay

Cells were collected in an ice-cold RIPA lysis buffer (P0013C, Beyotime, China) containing a protease inhibitor cocktail (P1006, Beyotime, China) in a microcentrifuge tube. After sonication (20–30 s) at 4°C and being placed in a centrifuge (10,000 rpm) for 5 min, the supernatant was used in the subsequent assays. Protein concentrations were determined according to the manufacturer’s instructions using a BCA protein assay kit (P0011, Beyotime, China). In brief, protein standard solutions were prepared with the following concentrations: 0, 0.025, 0.05, 0.1, 0.2, 0.3, 0.4, and 0.5 mg/ml of bovine serum albumin. A BCA working reagent was prepared by mixing solution A and solution B in a 50:1 ratio. In a 96-well plate, 20 μL of samples were mixed with 200 μL of BCA working solution (*n* = 3) and then incubated at 37°C for 2 h. The absorbance was measured using a microplate reader at 562 nm. The standard curve was used to determine the protein concentration of the samples.

### Western Blot

The collected samples were denatured by heating them for 5 min at 95°C with SDS-PAGE Sample Loading Buffer (P0015L, Beyotime, China). For SDS-PAGE, equal amounts of protein (20 g total protein) were loaded, and the gel was run at two stages: first 60 V for 20 min and then 80 V for 90 min using the Bio-Rad Mini-Protean (1,658,001). After activating the PVDF (FFP26, Beyotime, China) with methanol for a few minutes, the protein from the gel was transferred to the PVDF at 200 mA for 90 min using Bio-Rad Min Trans-Blot (1703930). A blocking buffer was used to block the membrane for 15 min at room temperature (P0231, Beyotime, China). According to the manufacturer’s recommended ratio, the first antibodies were diluted with dilution buffer (P0023A, Beyotime, China) and used for overnight membrane incubation at 4°C. The membrane was washed in TBST (P0023C, Beyotime, China) three times for 5 min each time and then incubated for 1 h at room temperature with the recommended dilution of conjugated secondary antibody in the dilution buffer (P0023D, Beyotime, China). Again, TBST was used to wash the membrane three times for 5 min each, and then HRP substrate (Millipore, WBKLS0500) was added to the membrane. Images were taken using the chemiluminescence Gel Doc (Bio-Rad Gel Doc/Chemi Doc Imaging System). The antibodies used are listed in Supplementary Table S3.

### Statistical Analysis

The number of replications for each experiment is indicated in the figures’ legend. The data were expressed with a mean value ±SEM. A one-way comparison ANOVA with Tukey’s post hoc test or *t*-test was performed to evaluate the statistical significance. Statistical significance was expressed as * (*p* < 0.05), ** (*p* < 0.01), or *** (*p* < 0.001).

## Results and Discussion

### Substrate Characterization

**FIGURE 1:**
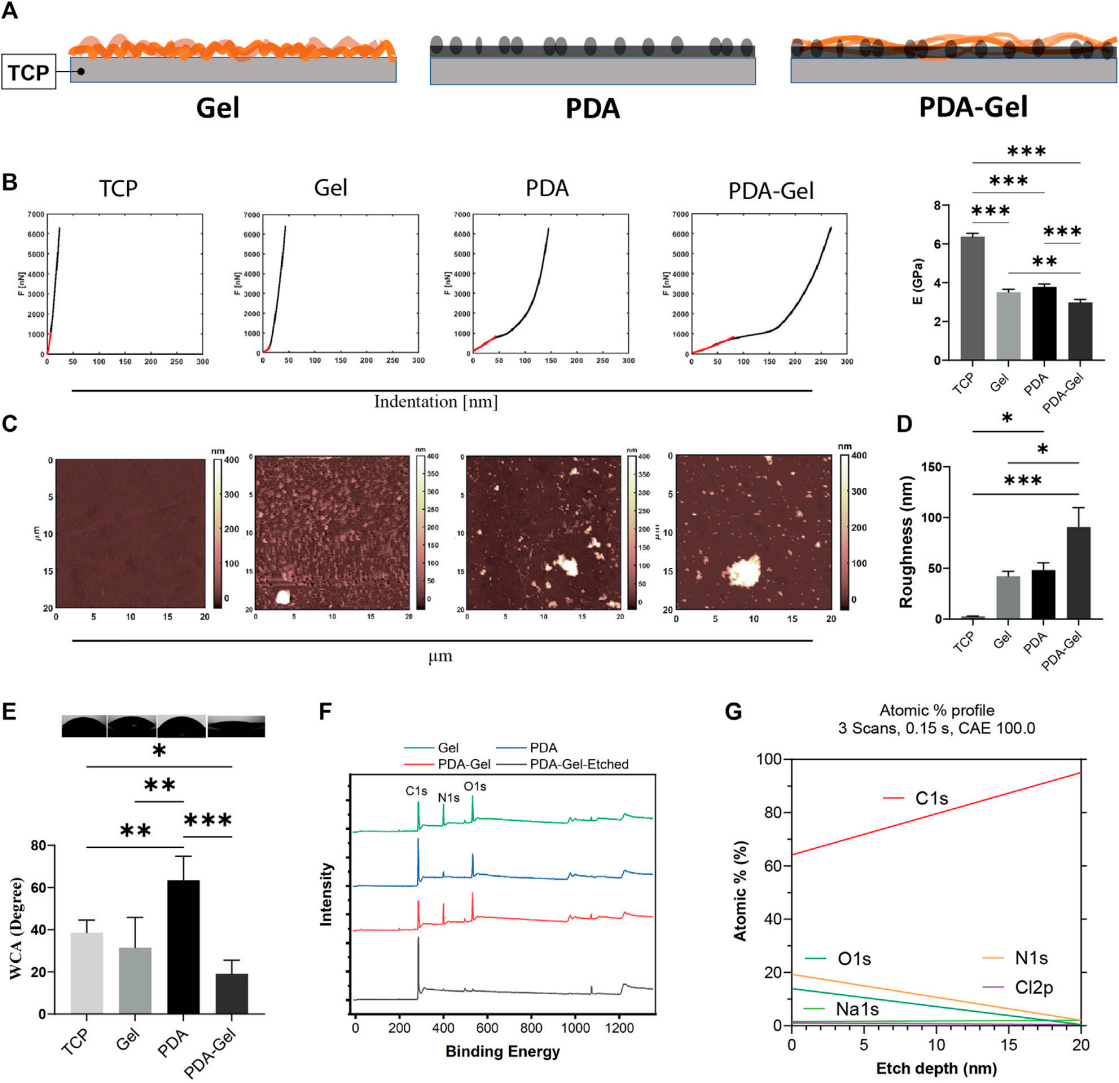
PDA effects on the surface characteristics. **(A)** Schematic representation of the substrates: Gel (gelatin), PDA (polydopamine), and PDA-Gel surfaces. The gel is physically adsorbed on the TCP while that is covalently bound to PDA. PDA is an underlayer facile conjugation of gelatin onto the surface by an amine group of lysine. **(B)** Nanoindentation for surface stiffness (force vs distance) and Young’s modulus (*n* = 2 for 4 < map). In the case of PDA data, it belongs to the coated film. Our observation showed that PDA particles have a lower Young’s modulus. **(C,D)** AFM analysis for surface topography and roughness. PDA particles with a thickness/size between 400 and 1000 nm (vertical) were seen on the PDA and PDA-Gel surfaces, respectively (*n* = 2 for 4 < map). **(E)** WCA measurement for surface wettability (*n* = 3). **(F)** XPS spectrum for surface elemental compositions. PDA-Gel-Etched means that the PDA-Gel surface coating was etched using an x-ray for 20 nm. **(G)** Depth profile of the elements. The PDA-Gel surface was etched 20 nm by XPS. Represented data and significance: mean ± SEM, ANOVA with Tukey test; *p < 0.05, **p < 0.01, ***p < 0.001.

Surface stiffness and roughness were analyzed using AFM on all substrates (i.e., TCP, Gel, PDA, and PDA-Gel surfaces) to examine the surface’s mechanical and structural properties (Figure 1A). The stiffness of the coatings has the following order: PDA-Gel < PDA ∼ Gel < TCP (Figure 1B). All measurements were carried out in DMEM to mimic cell culture conditions. The coating could be slightly swelled, resulting in a significant decrease in Young’s modulus from 6.3 ± 0.2 GPa (TCP) to 3.5 ± 0.1 GPa (Gel) (Figure 1B). On the PDA and PDA-Gel surfaces, Young’s modulus significantly decreased to 3.6 ± 0.1 GPa (PDA) and 2.9 ± 0.1 GPa (PDA-Gel), respectively. Considering the coatings are thin (∼20 nm) and the modulus of three types of coatings (PDA, Gel, and PDA-Gel) was still in the GPa range, the modulus represents the combined properties of the coatings and TCP that was only reduced by approximately 50% of the modulus. It is expected that PDA-Gel is the softest surface due to two consecutive coatings deposited on a harder substrate. The difference in the stiffness between the Gel and PDA-Gel surfaces could be due to a thicker coating of PDA-Gel layers and/or the presence of PDA aggregates on the surface. A previous study showed that PDA coating is nonuniform, which results in a heterogeneous stiffness distribution (Li et al., 2019). Our results also indicated a nonuniform mechanical map with the presence of aggregates (Supplementary Figure S1A).

PDA and PDA-Gel coatings produced heterogeneous roughness ranging from 48.0 ± 14.8 nm to 90.5 ± 38.3 nm, while Gel coating produced a more homogenous surface with a roughness of 42.0 ± 10.2 nm **(** Figures 1C,D **).** Note that we did not eliminate the adhesive effect between the AFM probe and samples. Surface roughness increases following PDA coating due to film and particle formation and aggregation (Kwon and Bettinger, 2018). The topographical map revealed that the PDA particles varied in height from 400 to 1,000 nm (Supplementary Figugre S1A). The surface roughness in this study was higher than in some previous studies (Sharma et al., 2019; Deng et al., 2021c), which may have contributed to the different protocols used. While many other studies analyzed a small area under dry conditions (1 μm^2^), our measurement was carried out in a medium over a 400 μm^2^ area. We deliberately tested a large area to show surface heterogeneities and particles.

Surface wettability, a critical feature in protein adsorption (Puliyalil et al., 2019) that subsequently regulates cell adhesion, has been found to improve following PDA coating by testing different substrate coatings of polystyrene (Cui et al., 2014). Because the TCP surfaces were treated with plasma before coatings, they exhibited hydrophilic characteristics where the WCA was 38.5° ± 6.0° (Figure 1E). PDA coating on the hydrophilic TCP raised WCA to 63.4° ± 11.0°, while Gel coating had relative wettability to the TCP (31.5° ± 7.0°). The PDA-Gel substrates have the lowest WCA (19° ± 2.0°), indicating that Gel has a higher amount and/or different structure between the PDA and TCP surfaces. While PDA can increase the hydrophilicity of a hydrophobic surface such as PDMS, it can also lower the wettability of a hydrophilic surface such as mica after plasma treatment (Sheng et al., 2015). As a consequence, PDA on the surface can keep the WCA within a specified range. Our findings also revealed that the top layer dominates the final wettability (i.e., gelatin).

The surface chemistry analysis using XPS showed that the nitrogen element (N%) on the Gel and PDA-Gel substrates (∼19%) was much higher than on the PDA substrate (∼6%, ∼3 times) due to the presentation of Gel (Figure 1F and Supplementary Figure S1B). The spectrums of Gel and PDA-Gel were similar, indicating that PDA-Gel was interwoven rather than two separated layers. We etched the PDA-Gel coating to see the depth-dependent elemental profile (Figures 1F,G). After etching by XPS, nitrogen signals were gradually decreased to about zero after 20 nm, indicating that the thickness of PDA-Gel in the dry state is about 20 nm. PDA concentration, pH, and the presence of oxygen during self-polymerization of PDA are determinants of PDA film properties such as roughness and thickness (Ball et al., 2012). Initially, we tried a PDA concentration of 2 mg/ml but found many aggregates on the surface that required a severe cleaning procedure (Lee et al., 2007). Then, we reduced the PDA concentration to 1.5 mg/ml for all experiments.

### Focal Adhesions

**FIGURE 2:**
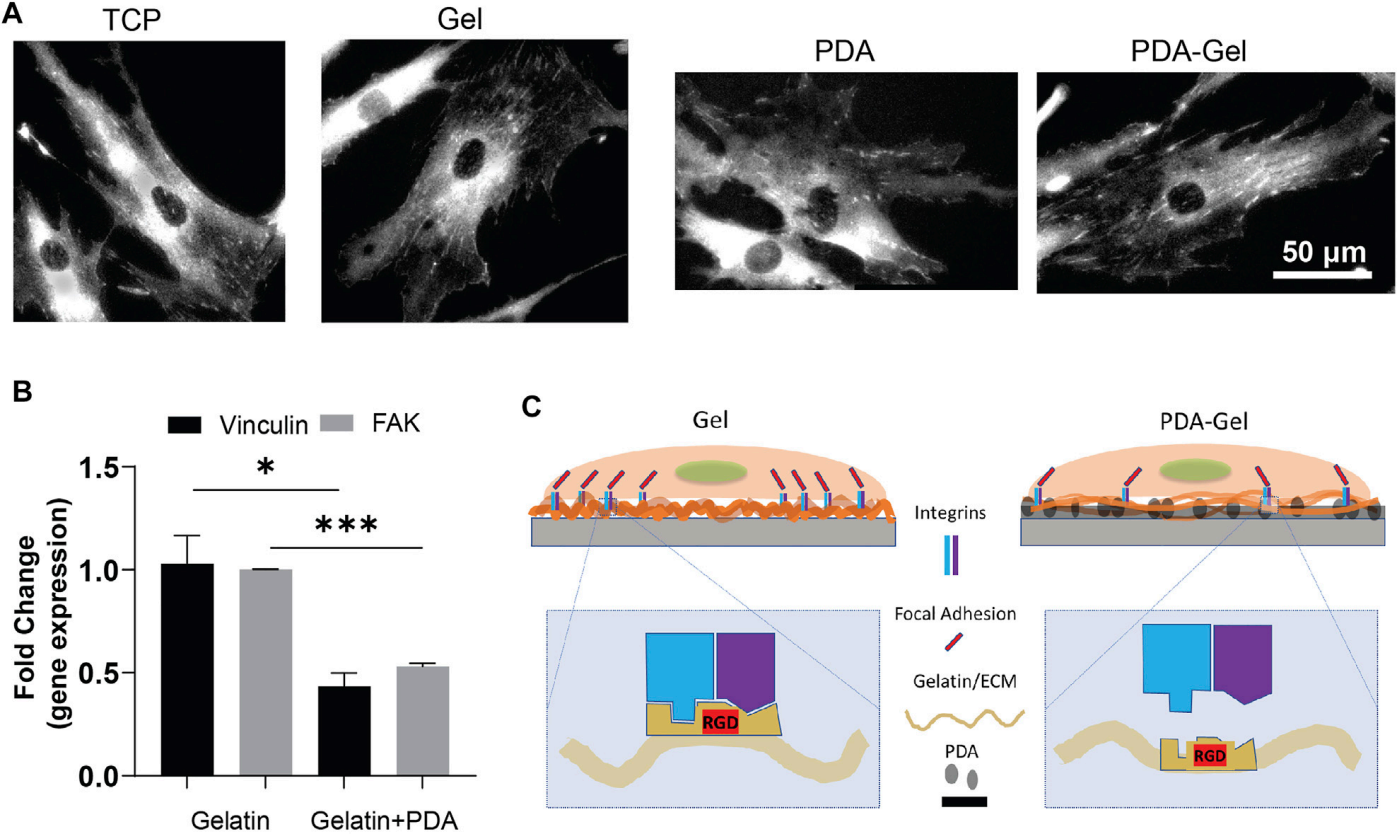
PDA effects on the focal adhesions of hASCs. **(A)** Vinculin staining of hASCs after cultivation for 24 h **(B)** qPCR results of vinculin and FAK (day 5, *n* = 3). Represented data and significance: mean ± SEM, *t*-test; *p < 0.05, **p < 0.01, ***p < 0.001. **(C)** Schematic representation of the change in expression of FAs after the presence of PDA as an underlayer of Gel. Higher vinculin and FAK expression on Gel than on PDA-Gel surfaces. When applying PDA as an underlayer coating, the conformation of gelatin is changed. Then, the exposed cell-binding sites are reduced.

FAs are important protein complexes for outside-in mechanotransduction (Teo et al., 2013; Burridge, 2017). The FA size is correlated to the ECM parameters such as stiffness and ligand density (Cao et al., 2017). Vinculin, a component of FAs, plays a crucial role in FA assembly and interconnect signaling to regulate integrin signaling for cell motility, growth, and differentiation (Kuo et al., 2011). The vinculin staining suggested that gelatin provided ligands for cell attachment and stimulated a prominent clustering of FAs complex (Figure 2A). Nevertheless, the immobilization of gelatin on the PDA (PDA-Gel) changes the features of gelatin, such as conformation, amount, and/or bioactivity. Alfieri et al. (2022) found that proteins attach to the PDA surface both physically and covalently, and the structural changes of proteins are likely to occur (Giol et al., 2015). The gene expression of vinculin and FAK revealed a 50% downregulation on the PDA-Gel substrate compared to the Gel surface (Figure 2B). Overall, PDA changes the gelatin adsorption and surface property resulting in lesser FAs on the PDA-Gel surface than on the Gel substrate. Downregulation of FAs causes a change in cell shape and probably a decrease in integrin signaling. A schematic illustration shows the difference between Gel and PDA-Gel (Figure 2C).

### Cell Migration

**FIGURE 3:**
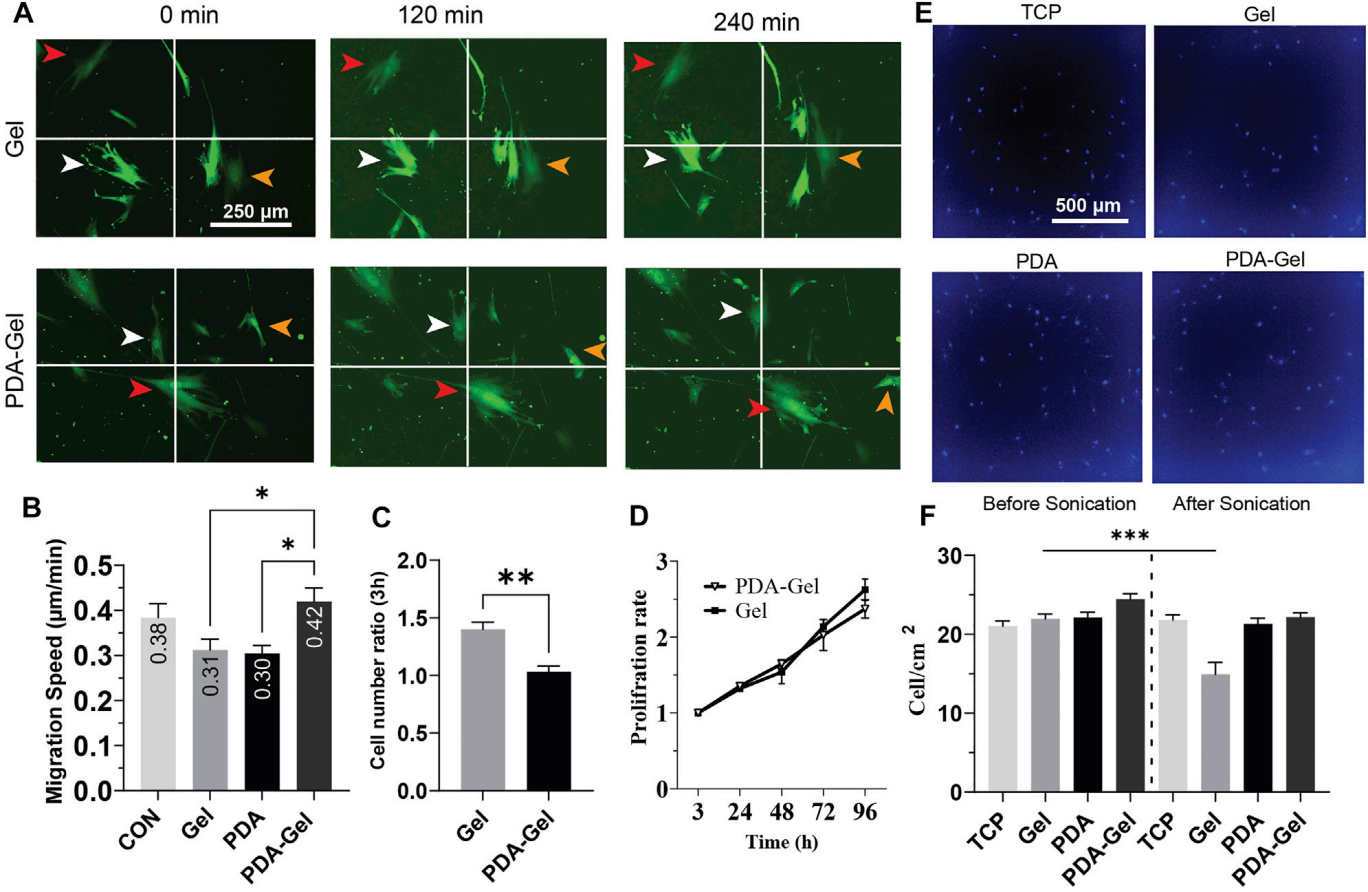
PDA effects on the proliferation and migration of hASCs **(A,B)** Cells were cultured overnight, and then images were captured at a 30-min interval for the analysis. Virally transduced GFP cells were used for this experiment. **(C)** Cell attachment after hASC seeding, the relative number of attached cells was measured by CCK-8 and normalized to the lowest number of absorbance (*n* = 3). **(D)** Cell proliferation was assessed by CCK-8 for 4 days and normalized for 3 h. **(E)** Representative images of hASCs after applying ultrasonication for 24 h. The nuclei were stained with Hoechst. **(F)** Cell density on each surface was compared before and after ultrasonication (*n* = 3). Represented data and significance: mean ± SEM, ANOVA with the Tukey test was used for the migration assay and *t*-test for cell attachment and ultrasonication *p < 0.05, **p < 0.01, ***p < 0.001.

The cell migration analysis demonstrated that neither gelatin nor PDA could alter cell migration speed alone or in combination with gelatin; however, compared to cells on Gel and PDA, the PDA-Gel substrate increased cell migration speed by roughly 1.4 times (Figures 3A,B). A recent study showed that a low concentration of PDA coating could boost MSC migration speed in response to fibronectin deposition and then integrin activation (Deng et al., 2021b). Given the link between FAs size and cell migration (Kim and Wirtz, 2013), PDA-Gel coating changes cell attachment and, consequently, changes the FA features (Figure 2), which results in faster cell movement.

### Cells Attachment and Proliferation

The initial cell attachment on the Gel substrate was approximately 1.5 times higher than that on the PDA-Gel substrate (Figure 3C). However, the proliferation rate between these two surfaces was no significant difference up to 96 h (Figure 3D). It indicates that both PDA and Gel are biocompatible for cell growth, but the PDA could alter gelatin properties leading to a lower initial cell attachment than the physically absorbed gelatin. Previous studies have observed that PDA improves the wettability of a hydrophobic surface and then promotes cell proliferation (Chuah YJ. et al., 2015; Deng et al., 2021c). In this study, PDA was applied on a hydrophilic TCP surface (WCA = 40°) and used for immobilizing and altering gelatin adsorption. Therefore, a lower initial cell attachment on PDA-Gel is no harm to using this coating for long-term stem cell culture. Gelatin facilitates cell attachment on the substrate by providing different ligands such as asparagine–glycine–aspartate (RGD), and any change in these binding motifs, such as conformation and structure of bioactive motifs, would affect cell attachment and proliferation (Davidenko et al., 2016).

Ultrasonication has been offered as an enzyme-free alternative approach for detaching adherent cells from the surface of the cell culture system (Kurashina et al., 2019). To acquire insights into substrates and ECM (coated gelatin/cell-secreted ECM) binding, we used ultrasonication to apply mechanical stress from below on both cells and ECM. Our results demonstrated that PDA-Gel coating maintains cell attachment under ultrasonication stress (Figures 3E,F), whereas cell–ECM detachment from the substrate was detected on the Gel substrate under the same circumstances, indicating that PDA improves the adhesion of cells by increasing the binding force of the proteins and ECM. PDA’s strong adhesive nature is attributed to the catechol and aminoethyl units found in PDA and then the reaction between the lysine (amine group) of protein and catechol (Lee et al., 2007). This finding suggests that ECM is tightly linked to PDA. Nevertheless, based on FA data, cells are most likely in a looser attachment state.

### hASC Differentiation

Lineage commitment of hASCs is strictly regulated by the ECM property, including physical and biochemical cues. After 5-day culture on the Gel and PDA-Gel substrates, hASCs were forced to differentiate into adipocyte-, osteocyte-, and chondrocyte-like cells using an induction medium. We investigated the level of differentiation, focal adhesions, and epigenetic state during differentiation. Therefore, the impact of PDA coating on stem cell differentiation can be explored.

### Adipogenic Differentiation

**FIGURE 4:**
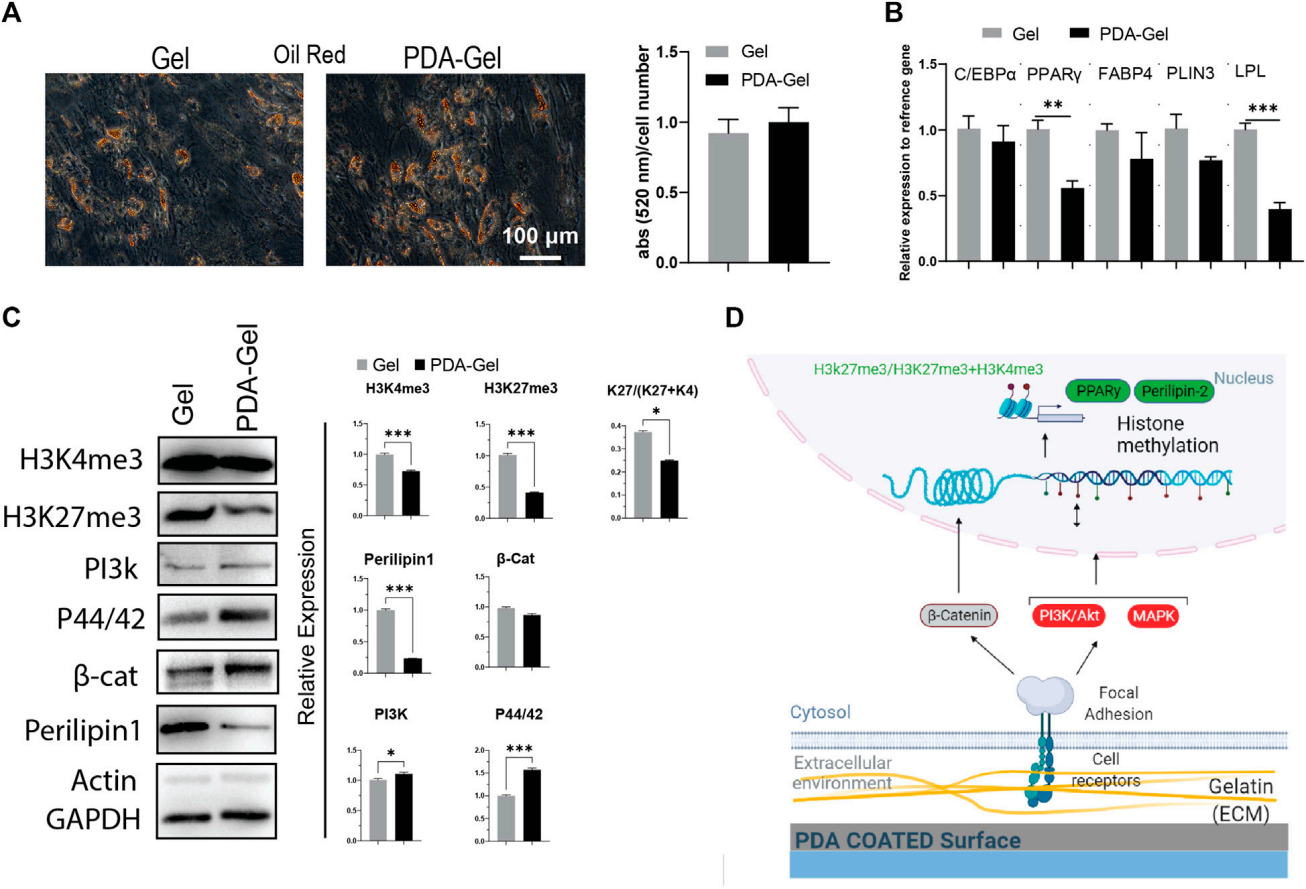
PDA effects on the adipocyte differentiation of hASCs. **(A)** Oil Red O staining after 7 days of hASCs adipocyte induction and the quantification of the extracted dye. **(B)** Gene expression analysis of adipocyte-related markers: C/EBP, PPARγ, FABP4, PLIN3, and LPL (*n* = 3). **(C)** Western blot results and semi-quantification of the signaling pathways and epigenetic markers. H3K27me3/(H3K27me3+H3K4me3): K27/(K27 + K4). **(D)** Possible mechanism of the PDA’s effects on adipocyte differentiation of hASCs. Represented data and significance: mean ± SEM, *t*-test, *p* < 0.05, **p < 0.01, ***p < 0.001.

Oil red staining after 7 days of adipogenic differentiation showed that both Gel and PDA-Gel substrates were equally effective, indicating that PDA coating could not improve adipogenic differentiation (Figure 4A). In addition, the gene expression analysis (Figure 4B) showed downregulation of PPARγ, one of the master regulators of adipogenesis, and lipoprotein lipase (LPL), an early marker of adipogenic differentiation with a transcription regulatory role. This finding might be related to changes in adipocyte development stages, which result in varied morphologies in oil droplets (Supplementary Figure S2 **)**. On PDA-Gel, oil droplets look to be more accumulated on one side of the nucleus, but on the Gel substrate, oil droplets seem to be more randomized. There are no changes in other genes, such as C/EBPα, FABP4, and PLIN3, during adipogenic differentiation, resulting in the same differentiation efficiency (lipid production) on both substrates (Lefterova et al., 2008). Adipogenic differentiation of hASCs is influenced by surface properties such as stiffness (Young et al., 2013; Zhang et al., 2018). Here, PDA coating changes surface stiffness and also other properties, which leads to a fluctuation of gene expression in the adipocyte development stage.

Our results confirmed that the PDA-Gel substrate affected adipogenic differentiation by demonstrating a reduced expression of Perilipin 1 (PLIN1) protein levels (approx. 4 times), a biomarker of the mature stage of adipogenesis (Itabe et al., 2017). Mechanotransduction signaling pathways (PI3K and MAPK) were increased following PDA coating, but there was no alteration in the WNT (β-catenin) pathway (Figure 4C). A prior study found that inhibiting PI3K and MAPK *via* miRNA regulation reduced adipogenesis (Wang et al., 2020). Induction of PI3K may not be sufficient to improve adipogenic differentiation, or it may interact with other signaling pathways, such as MAPK, to regulate the adipocyte development stage.

We also investigated the epigenetic landscape as part of the cellular response to PDA coating using two chromatin condensation markers, H3k27me3 and H3k4me3, which are normally linked with downregulation and upregulation of gene expression, respectively (Liu et al., 2016; Stachecka et al., 2021). Both markers were found to be expressed at a lower level on the PDA-Gel surface than on the Gel surface. More importantly, the ratio of H3k27me3-to-H3k4me3+H3K27me3 was lower on the PDA-Gel surface (Figure 4C). The ratio is a key indicator of chromatin status, an important element in cell fate decisions through gene expression regulation (de Gobbi et al., 2011). PDA coating could have a negative effect on the adipogenic differentiation stage. A possible mechanism for the influence of the PDA-Gel substrate on adipogenic differentiation is illustrated **(** Figure 4D **)**.

### Osteogenic Differentiation

**FIGURE 5:**
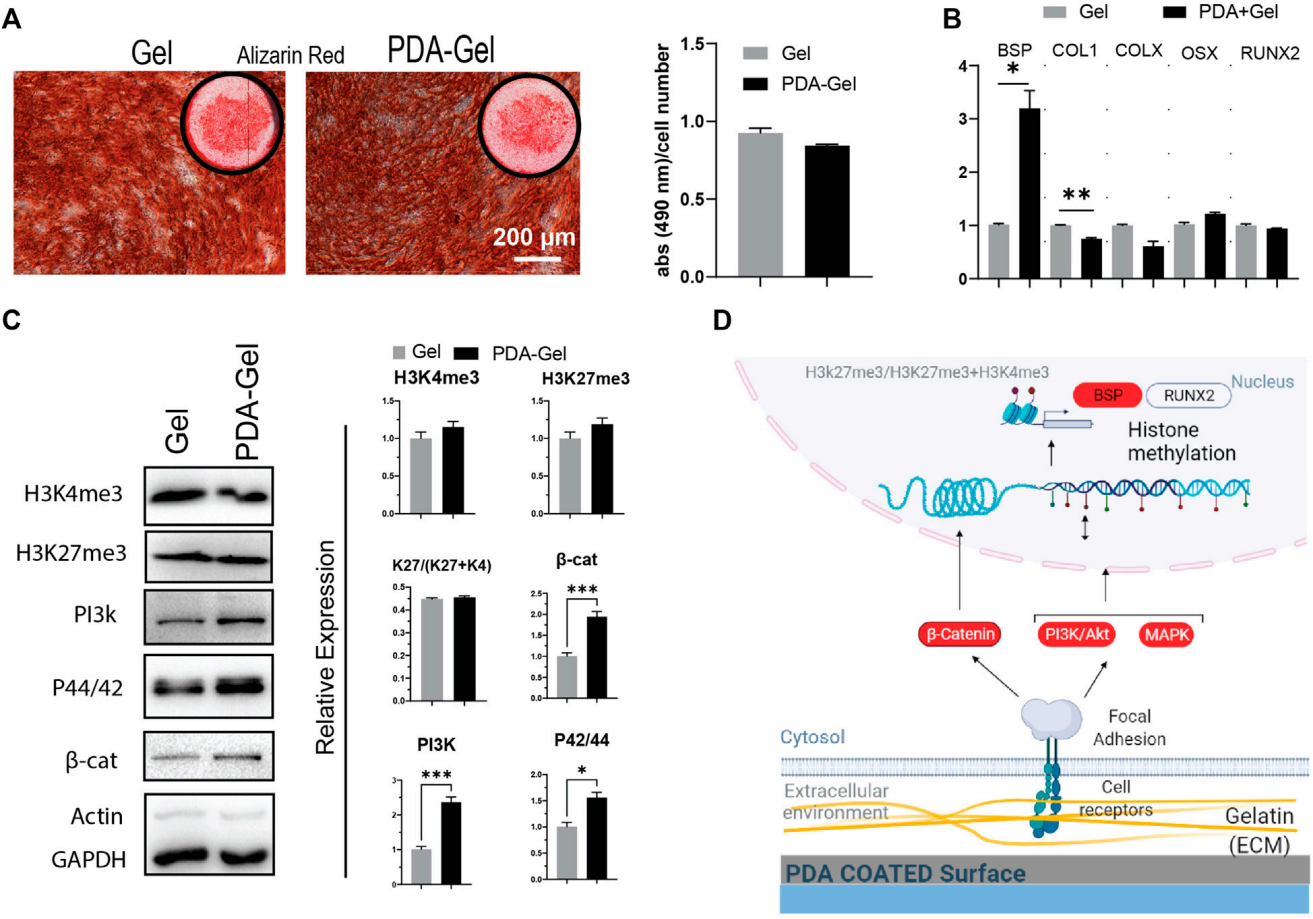
PDA effects on the osteocyte differentiation of hASCs. **(A)** Alizarin Red staining after 21 days of osteogenic induction. Quantification of the extracted dye. **(B)** Gene expression analysis of osteocyte-related markers: BSP, COL1, COLX, OSX, and RUNX2 (*n* = 3). **(C)** Western blot results and semi-quantification of the signaling pathways and epigenetic markers. H3K27me3/(H3K27me3+H3K4me3): K27/(K27 + K4). **(D)** Possible mechanism of the PDA’s effects on osteocyte differentiation of hASCs. Represented data and significance: mean ± SEM, *p < 0.05, **p < 0.01, ***p < 0.001.

The AR staining revealed no significant differences between the PDA-Gel and Gel substrates (Figure 5A). AR staining, on the other hand, indicated changes in calcium deposition morphology, as marked cells on Gel are smoother and more uniform (Figure 5A **)**. Assessment of different osteogenic genes only showed higher expression of bone sialoprotein (BSP, ∼3 times) after 21 days (Figure 5B), a non-collagenous integrin-binding protein accompanied by pre-osteoblasts transformed to fully differentiated osteoblasts (Miron and Zhang, 2012). We also checked the expression of genes (Runx2, ALP, BSP, COL1, and OSX) at earlier time points (7 days); however, it again indicates no significant changes (Supplementary Figure S3).

The epigenetic markers, H3K4me3 and H3Kme27, did not differ substantially between the PDA-Gel and Gel substrates (Figure 5C). Considering the signaling pathways regulating osteogenesis by mechanical stimuli, PI3K, MAPK, and β-catenin (Marie et al., 2014; Song et al., 2017), all three markers were shown to be upregulated after 21 days of induction. Despite changes in the signaling pathway expression, the absence of significant alterations in the epigenetic ratio may explain the comparable pattern in gene expression and, as a result, differentiation outcomes. This finding implies that differences in signaling pathways may be attenuated by the final observed epigenetic state. Previous research suggests a relationship between ECM biophysical signals such as roughness and stiffness and osteogenic differentiation efficiency (Wang et al., 2016; Leach and Whitehead, 2018; Sun et al., 2018). The reason for our observed results for the similar efficiency of both substrates in osteocyte development would be that the cells produced ECM proteins during osteocyte induction and thus modified the microenvironment, perhaps attenuating the action of PDA. Another possible reason is that the sensitivity of hASCs to biophysical stimuli varies depending on the donor (Sundelacruz et al., 2015). Altogether, PDA coating could have no effect on the osteogenic differentiation stage. A possible mechanism for the influence of the PDA-Gel substrate on osteogenic differentiation is depicted (Figure 5D.)

### Chondrogenic Differentiation

**FIGURE 6:**
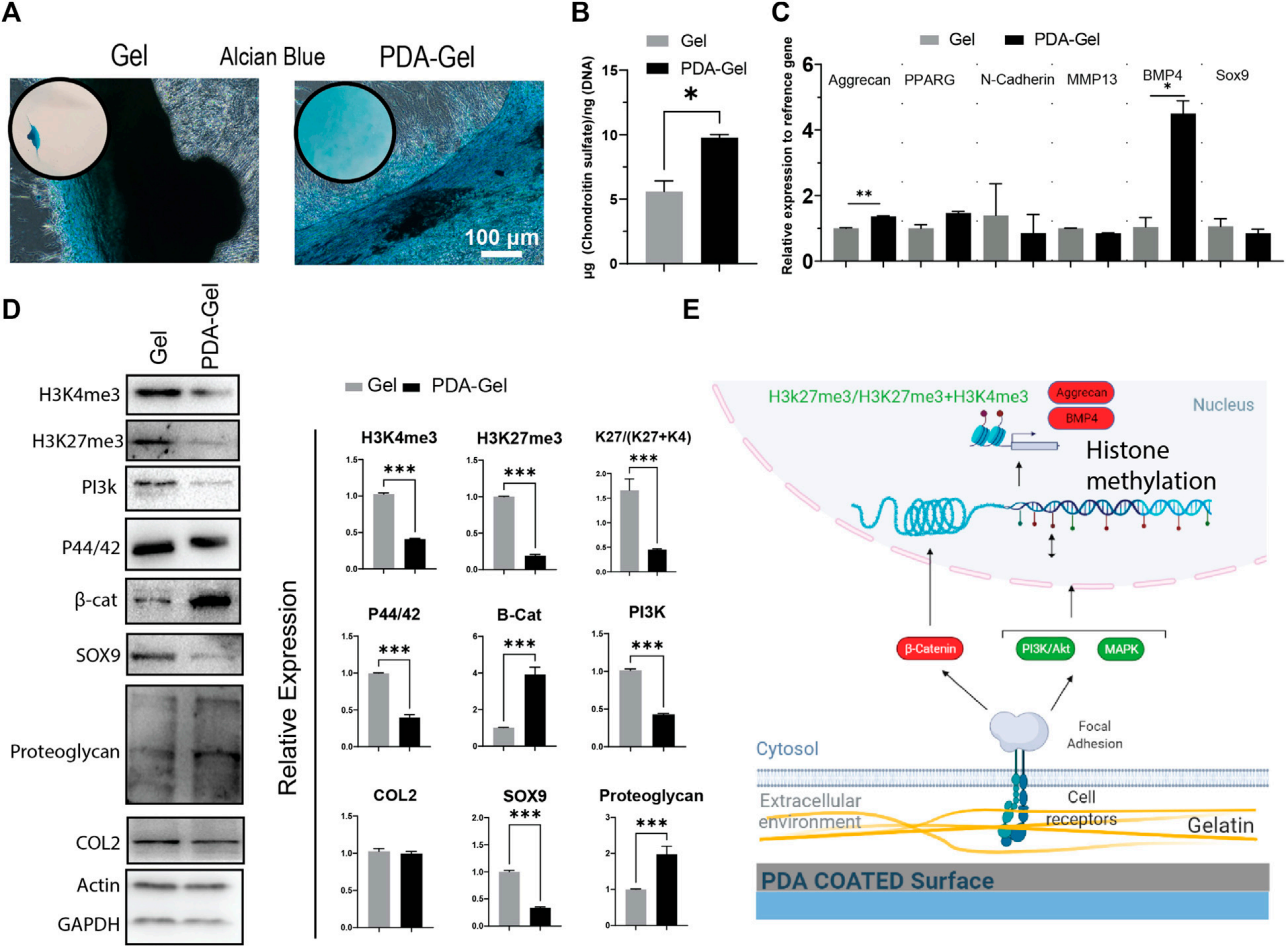
PDA effects on the chondrocyte differentiation of hASCs. **(A)** Alcian Blue staining after 21 days of chondrogenic induction. **(B)** Quantification of chondroitin sulfate by GAG assay. **(C)** Gene expression analysis of chondrocyte-relatedmarkers: aggrecan, PPARG, N-cadherin, MMP13, BMP4, and Sox9 (*n* = 3). **(D)** Western blot results and semi-quantification of the signaling pathways and epigenetic markers. H3K27me3/(H3K27me3+H3K4me3): K27/(K27 + K4). **(E)** Possible mechanism of the PDA’s effects on chondrocyte differentiation of hASCs. Represented data and significance: mean ± SEM, *p < 0.05, **p < 0.01, ***p < 0.001.

After 21 days of induction, Alcian Blue staining was employed to confirm hASCs chondrogenic development. By keeping cells in contact with the substrate, the PDA-Gel substrate inhibited cell aggregation (Figure 6A). This effect, when paired with the ultrasonication data (Figures 2E,F), demonstrates that PDA coating increases ECM attachment stability, hence stabilizing the cells on the surface during chondrogenic induction. A similar effect has been reported previously when a PDA coating was applied to an implanted material; the consequence was an increase in the substance’s integration force with the tissue (Dinh et al., 2018). The change from 2D morphology to cell aggregates (3D) at an early stage of chondrocyte induction might be a critical parameter in the final results, as cells on the Gel substrate would not be in contact with the substrate for more than 10 days. Our findings are in the same line as previous research that has shown a soft substrate with weaker cell adhesion strength may improve chondrogenesis of MSCs (Park et al., 2011).

Chondrogenesis is a morphogenetic process that involves the proliferation, condensation, and differentiation of MSCs into chondrocytes, followed by maturation into hypertrophic chondrocytes (Green et al., 2015). Changes in substrate parameters, such as reduced stiffness, increased hydrophobicity, and roughness, have been demonstrated to influence chondrogenesis (Chuah Y. J. et al., 2015). The PDA-Gel substrate stimulated GAG (glycosaminoglycan) formation almost twice as much as the Gel substrate (Figure 6B), which is substantiated by overexpression of BMP4 (a polypeptide that triggers chondrogenesis) and aggrecan (major proteoglycan in articular cartilage) gene (Figure 6C). Furthermore testing on the protein markers revealed that cartilage proteoglycan expression was higher (about 3-fold higher) and Sox 9 expression was lower (about 3-fold lower) as a stage-dependent transcription factor during differentiation (Chen et al., 2021), suggesting variations in the differentiation stage (Figure 6D). Nevertheless, collagen 2, a component of cartilage tissue, showed no change between the substrates, indicating that the cell subtype has not been defined.

The mechanistic investigation was shown a higher level of canonical WNT/β-catenin (about 4-fold) with a role in chondrocyte maturation (Dao et al., 2012), supporting the hypothesis that PDA facilitates or accelerates hASC differentiation to chondrocytes. ERK1/2 (P44/42) and PI3K, on the other hand, were expressed at a lower level and are important in the early stages of differentiation (Kita et al., 2008; Matta and Mobasheri, 2014). Both H3K4me3 and H3Kme27 were downregulated, and the ratio of H3K27me3-to-H3Kme27 + H3K4m3 on the PDA-Gel surface was reduced about three-fold, showing that the hASCs had relatively open chromatin, which may have assisted chondrocyte growth in the adherent state. Altogether, PDA coating could improve chondrogenic differentiation. The impact of PDA-Gel on hASCs chondrogenesis is summarized (Figure 6E).

### Integrins and Syndecans

**FIGURE 7:**
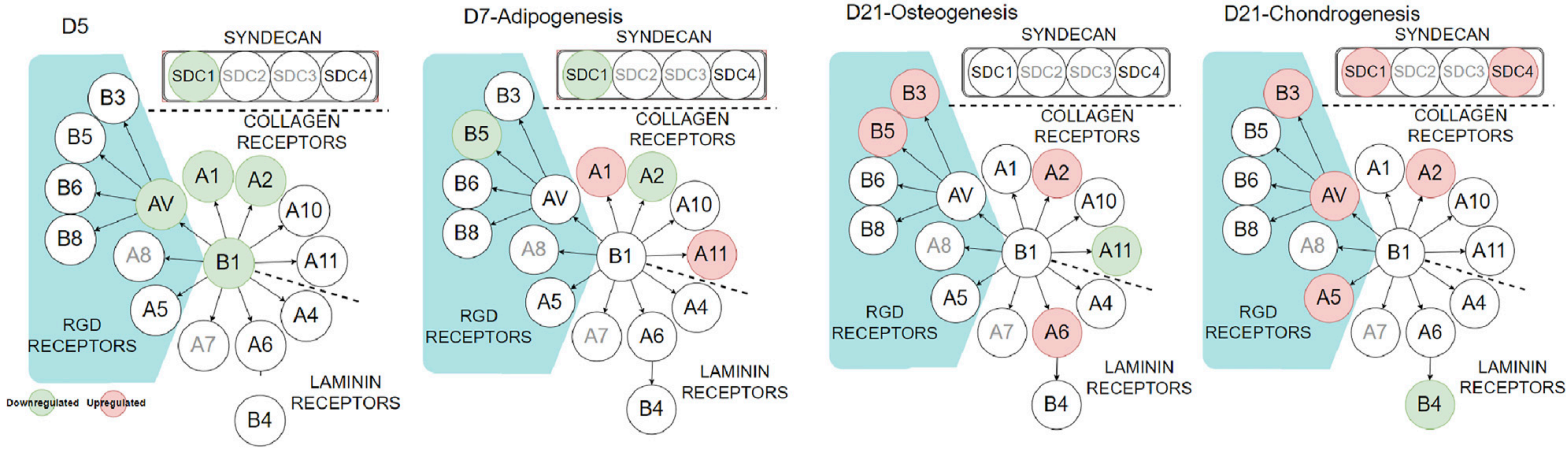
Integrin and syndecan expressions at different stages. Red and green circles indicate up- and downregulation of the corresponding genes (PDA-Gel vs. Gel). Gray circles were not considered in this study.

To further investigate the influence of PDA on cell–ECM interaction, we looked at integrins and syndecans (SDCs), cell surface receptors that transform external stresses into internal biological signals, a process known as mechanotransduction. SDCs are a small family of transmembrane proteoglycans that have a function in mechanotransduction (Ibrahim et al., 2012; Le et al., 2018). They have been linked to the regulation of integrin α2β1 (Vuoriluoto et al., 2008) and αvβ3 (Beauvais et al., 2004). The cell receptors were analyzed using qPCR during cell expansion (day 5) and differentiation stages (day 7 or day 21, Figure 7 and Supplementary Figure S4). During the cell-expansion stage, as expected by the FA results (Figure 2), several integrins and SDC1 were downregulated (green circles) or had no difference on the PDA-Gel substrate compared to the Gel surface. The downregulation of integrin α2 by more than half and integrins α1, β1, αV, and SDC1 by about half on the PDA-Gel substrate would have correlated with the corresponding reduction in the FA expression (Figure 2).

**FIGURE 8:**
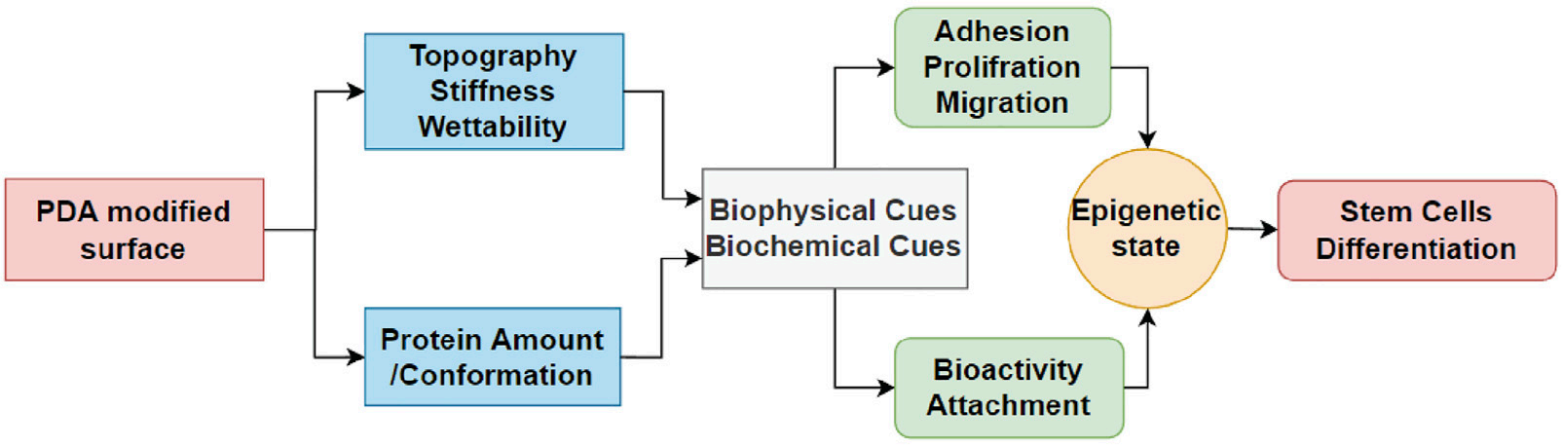
Schematic flowchart representing how PDA coating affects the surface and then differentiation of hASCs. For the first time, the effect of PDA on the epigenetic state of stem cells is reported in this study, which is crucial for biomaterials and stem cells.

Interestingly, the expression of cell receptors was upregulated (red circles) after differentiation, depending on the lineage. For example, integrins α2 and α11 (collagen receptors) were upregulated in adipogenic cells, while α2 and β3 were upregulated in both osteogenic and chondrogenic cells on the PDA-Gel substrate. In the case of chondrogenesis, β4 (laminin receptor) was downregulated, while α2, α5, αV, β3, SDC1, and SDC4 were upregulated. We hypothesize that cell attachment to the PDA-modified ECM/gelatin with integrins and SDC during the expansion stage plays an important role in cell destiny regulation. Because the cell microenvironment is transformed subsequently by cell-secreted new ECM, it cannot be a perfect indicator for judgment, even if the new ECM would have been developed as a result of the initial triggers. In the expansion stage, our results for integrin downregulation support another study that found an inhibitory impact of ITGBL1 in chondrogenesis promotion as well as evidence for the integrin-α5β1 inhibitory role in cartilage development (Song et al., 2018). Other investigations have also found that integrin activation has an inhibitory impact on chondrogenesis (Takahashi et al., 2003; Connelly et al., 2007). The discovery of the association between the downregulated state of integrins and lineage differentiation to adipocytes supports earlier findings by demonstrating the promoting effect of inhibiting integrins β1 and αV in adipocyte differentiation (Chen et al., 2014). Surface structure, mechanics, and protein adsorption are all affected by PDA precoating. Cellular adaptation, particularly varied expression of the cell receptors such as integrins and SDCs, functions as determinants, initiating a cascade of modifications that convert PDA-induced biophysical inputs into biological signals. Overall, hASCs exhibit distinct features in terms of the differentiation stage of development, which is supported by a changed epigenetic state. Figure 8 displays a schematic representation of our suggested model for modulating ASC differentiation *via* PDA-modified surface differentiation (as an underlayer of protein coating). Notably, our findings have sparked a new line of inquiry into how the PDA surface coating influences the protein-accessible motif for cell attachment or the preferred binding arrangement of proteins to PDA.

## Conclusion

PDA is a well-known cell-adhesive polymer for biomedical device surface modification. However, as a coating material, it does not have obvious biological effects but has a dominant effect on protein adsorption and ECM adhesion. Our findings highlight the significance of PDA–protein interaction, thus altering stem cell culture and differentiation. PDA coating causes changes in surface topography, stiffness, and protein adsorption, which in turn affect hASC behaviors such as FA, attachment, and migration. These cellular behaviors alter the gene expression and epigenetic state of hASCs during lineage differentiation, especially chondrogenic differentiation. Thus, PDA coating (PDA-Gel vs. Gel) has a positive effect on chondrogenic differentiation, while there is a limited effect on adipogenic and osteogenic differentiation. More importantly, we explore a possible mechanism where the epigenetic state of hASCs during chondrogenic differentiation was significantly modulated on PDA-Gel. For the first time, this in-depth study provides new insights into the effect of PDA coating on stem cells.

## Data Availability

The original contributions presented in the study are included in the article/Supplementary Material; further inquiries can be directed to the first and corresponding authors.
