# Supplementary material for: Polydopamine-Mediated Protein Adsorption Alters the Epigenetic Status and Differentiation of Primary Human Adipose-Derived Stem Cells (hASCs)
Source: Front Bioeng Biotechnol. 2022 Aug 10;10:934179. doi: 10.3389/fbioe.2022.934179 (PMC9399727; doi:10.3389/fbioe.2022.934179)
Supplement: Supplementary file 1 [file DataSheet1.docx]

Supplementary file

**Polydopamine-mediated protein adsorption alters the epigenetic status and differentiation of primary human adipose stem cells (hASCs)**

**Javad Harati^1,2,3^, Xuelian Tao^1^, Hossein Shahsavarani^4^, Ping Du^1^, Massimiliano Galluzzi^5^, Kun Liu^1^, Zhen Zhang^1,2^, Peter Shaw^6^, Mohammad Ali Shokrgozar^3^, Haobo Pan ^1,*^, Peng-Yuan Wang ^1,6,*^**

^1^Shenzhen Key Laboratory of Biomimetic Materials and Cellular Immunomodulation, Shenzhen Institute of Advanced Technology, Chinese Academy of Sciences, Shenzhen, Guangdong, China

^2^University of Chinese Academy of Sciences, Beijing, China

^3^Lab Regenerative Medicine and Biomedical Innovations, Pasteur Institute of Iran, Tehran, Iran

^4^Dept. Cell and Mol Biol, Faculty of Life Science and Biotechnology, Shahid Beheshti Univ., Tehran Iran

^5^Materials Interfaces Center, Shenzhen Institutes of Advanced Technology, Chinese Academy of Sciences, Shenzhen, Guangdong, China

^6^Oujiang Laboratory; Key Laboratory of Alzheimer's Disease of Zhejiang Province, Institute of Aging, Wenzhou Medical University, Wenzhou, Zhejiang, China

*** Correspondence:**Corresponding Author
Peng-Yuan Wang: [py.wang@ojlab.ac.cn](mailto:py.wang@ojlab.ac.cn); Haobo Pan: [hb.pan@siat.ac.cn](mailto:hb.pan@siat.ac.cn)

Keywords: polydopamine, adipose stem cells, integrin, chondrogenic, epigenetic state

Fig. S1

**Figure. S1**| (a) The morphology and stiffness maps of different substrates measured by AFM (b) atomic percentage of the elements on the different substrates


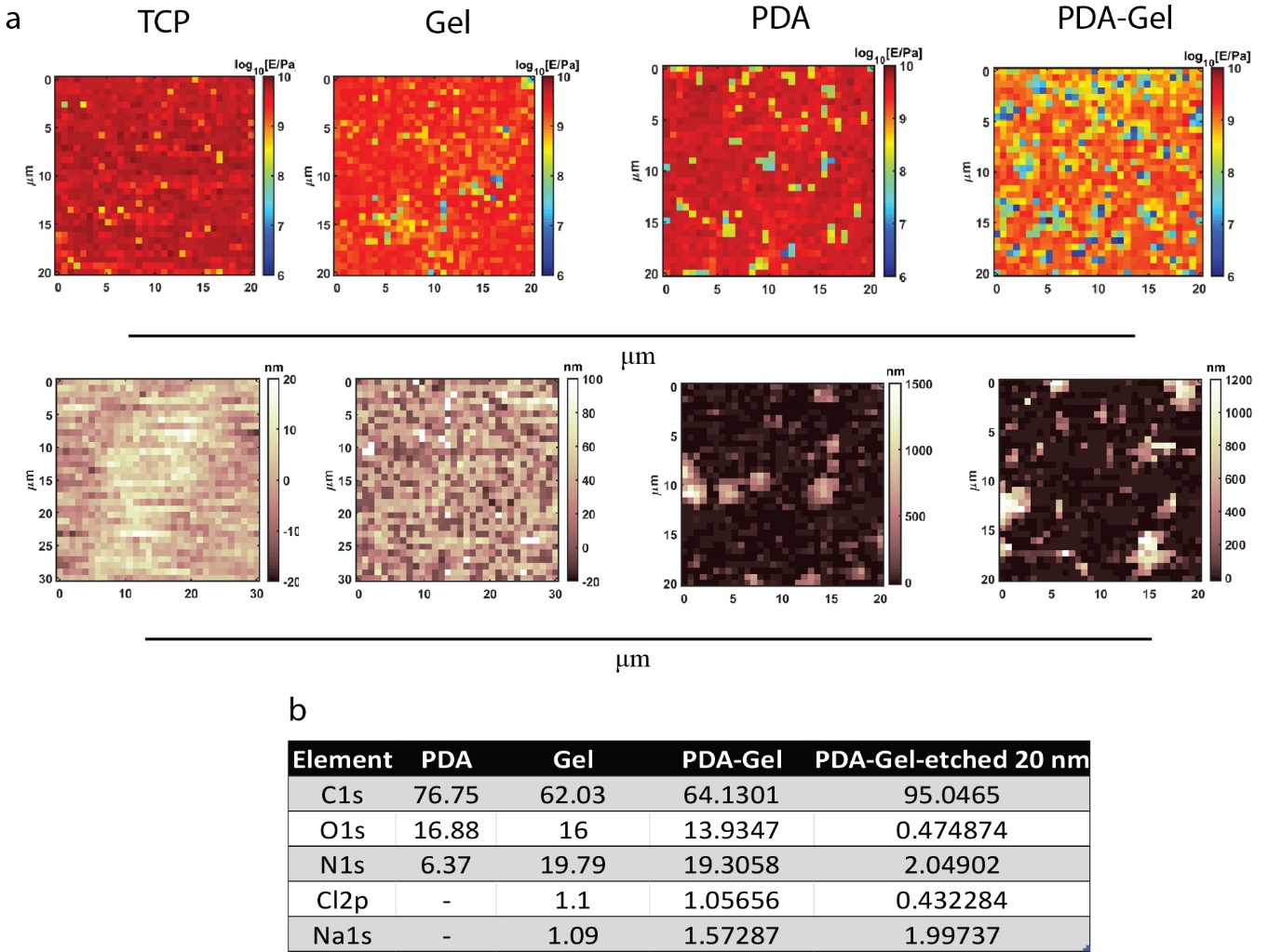


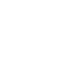


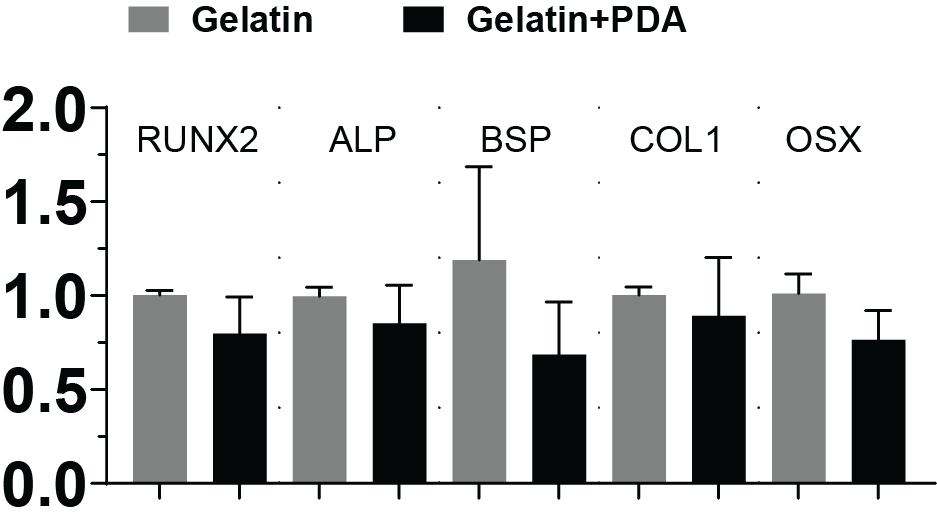
Fig. S2

**Figure. S2|** The morphology of oil droplets accumulation on day 7 after ASCs' differentiation induction to adipocyte**.**


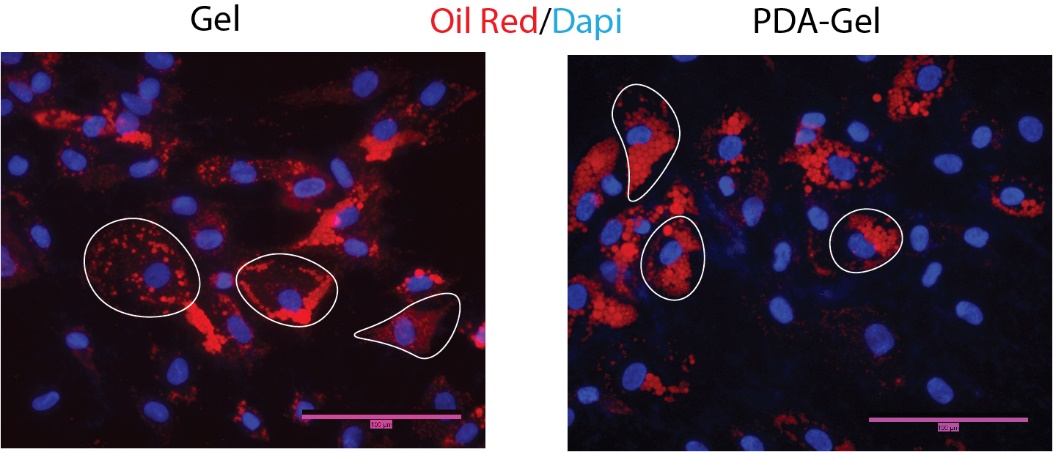


Fig. S3


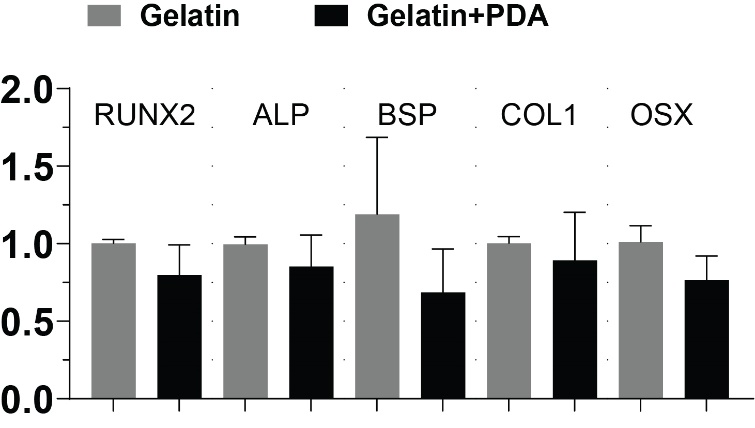


**Figure. S3|** The gene expression analysis of osteocyte related markers on day 7 post induction**.**

Fig. S4


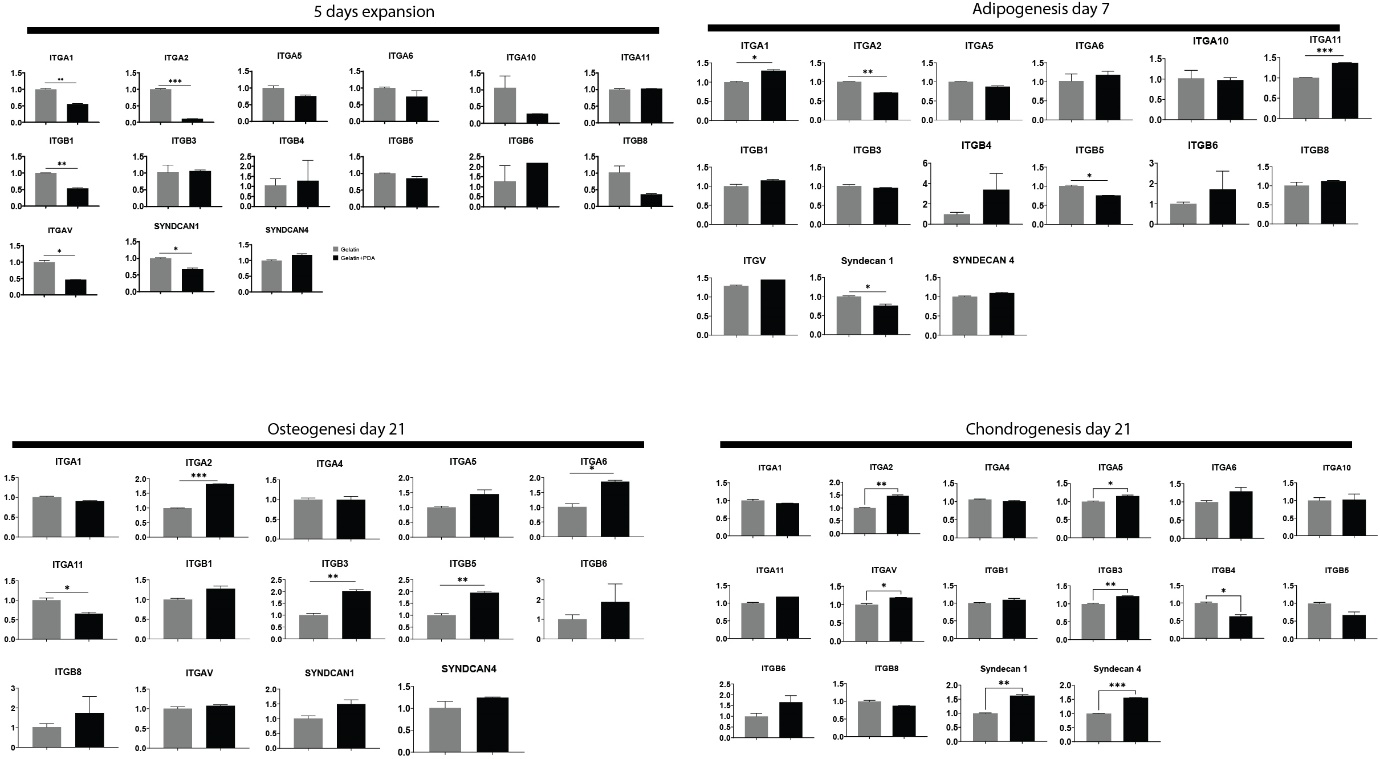


**Figure. S4|** The qPCR results of the cell receptors study (abstract graph in Fig. 7a)

**Table S1**. Media formulations used for differentiation study

| **Chondrogenesis reagent** | **Concentration** | **Catalog No.** |
| --- | --- | --- |
| Dexamethasone | 0.05 μM | Sigma: D8893-1mg |
| Ascorbate-2-phosphate | 25ng/ml | Sigma: A8960-5g |
| L-Proline | 20μg/ml | Sigma: P5607-25g |
| Sodium pyruvate | 50 μg/ml | Sigma: S8636 |
| ITS (Insulin Transfer Selenium) | 0.5% | BD: 354352 |
| BSA | 62.5 mg/ml | YEASEN |
| TGF-β1 | 5 ng/ml | PROSPEC |
| Penicilin/streptomycin | 1% | Gibco |
| FBS | 1% | Gibco |
| DMEM | - | Gibco |
| **Osteogenesis induction reagent** |  |  |
| Dexamethasone | 0.05 μM | Sigma: D8893-1mg |
| Ascorbate-2-phosphate | 25ng/ml | Sigma: A8960-5g |
| β-glycerophosphate | 5 mM | Sigma: 50020/G9422 |
| Penicilin/streptomycin | 1% | Gibco |
| FBS | 10% |  |
| MEM-alpha | - | Gibco |
| **Adipogenesis induction reagent** |  |  |
| Dexamethasone | 0.05 μM | Sigma: D8893-1mg |
| IBMX | 250 μM |  |
| Insulin | 5 mg/ml | Gibco |
| Indomethacine | 100 μM |  |
| Penicilin/streptomycin | 1% | Gibco |
| FBS | 10% | Ginco |
| MEM-alpha | - | Gibco |

**Table S2.** List of primers used in qPCR

| **Name** | **Forward** | **Reverse** |
| --- | --- | --- |
| h-ITGA5 | ATCTGTGTGCCTGACCTG | AAGTTCCCTGGGTGTCTG |
| h-ITGB3 | AATGCCACCTGCCTCAAC | GCTCACCGTGTCTCCAATC |
| h-ITGA6 | GAGCTTTTGTGATGGGCGATT | CTCTCCACCAACTTCATAAGGC |
| h-ITGB4 | GCTTCACACCTATTTCCCTGTC | GACCCAGTCCTCGTCTTCTG |
| h-ACTB | CACCATTGGCAATGAGCGGTTC | AGGTCTTTGCGGATGTCCACGT |
| h-ITGB5 | CTCGGAGCCCAAGTCGC | GGACTCCTAGTGTGTCGCC |
| h-ITGA10 | TGAACATCACCCACGCCTATTCC | ATCCATGTATGTTGGGCAGCCAT |
| h-ITGA4 | TCCATGCTTCCTCCATAAAGAC | ACAGCTCTGTTGGGAATGCT |
| h-ITGB6 | GAGGGGAAAACCATCATTCA | GTAGGACAACCCCGATGAGA |
| h-Col I-F | CTGGATGCCATCAAAGTCTTC | AATCCATCGGTCATGCTCTC |
| h-Col X-F | TGGGACCCCTCTTGTTAGTG | TTGGGTCATAATGCTGTTGC |
| h-Runx2-F | TGCACTGGGTCATGTGTTTG | TGGCTGCATTGAAAAGACTG |
| h-BMP4 | TTGTCTCCCCGATGGGATTC | TACGGAATGGCTCCATAGGT |
| h-Aggrecan-F | TCTGTAACCCAGGCTCCAAC | TGGAGTACCTGGTGGCTCTC |
| h-SOX9-F | AAAGGCAAGCAAAGGAGATG | TGGTGTTCTGAGAGGCACAG |
| h-MMP13-F | AACATCCAAAAACGCCAGAC | GCATCAATACGGTTGGGAAG |
| h-N-cadherin-F | GATCAGCGTGCTGGACGTAGAT | TGGTCCCTACCGGAGTGTCTTC |
| h. NRCAM-F | CCCTGATTCTCTTCCTGTGCC | GTTGGAGGCTGTACCAAGTC |
| h.FAK-F | CGTCTAATCCGACAGCAACA | ACTGCCTCGAGAGAGTCTCACAT |
| hPlin3 | GCCCAAGAGATGGTGTCTAGC | CCGGTCACTACGGACTTTGT |
| h-LPL-F | GACTCGTTCTCAGATGCCCTACAA | CCACCAGTCTGACCAGCTAAAGTA |
| h-BSP | CACTGGAGCCAATGCAGAAGA | TGGTGGGGTTGTAGGTTCAAA |
| h-PPARγ-F | AGCCTCATGAAGAGCCTTCCA | TCCGGAAGAAACCCTTGCA |
| h-C/EBPα-F | CTTGTGCCTTGGAAATGCAA | GCTGTAGCCTCGGGAAGGA |
| h-FABP4-F | ACTGGGCCAGGAATTTGACG | CTCGTGGAAGTGACGCCTT |

**Table S3**. List of antibodies used in this study

| **Antibody** | **Supplier** | **Cat. number** |
| --- | --- | --- |
| GAPDH | Cell signaling | 14C10 |
| H3K4me3 | abcam | ab8580 |
| H3k27me27 | abcam | ab6002 |
| B-catenin | Cell signaling | 8480 |
| PI3 Kinase p110α | Cell signaling | 4249 |
| P42/44 | Cell signaling | 4695 |
| Perilipin1 | Cell signaling | 9349 |
| Sox9 | abcam | ab76997 |
| Anti-Cartilage-Proteoglycan | Millipore | Mab2015 |
| Col-2 | abcam | ab188570 |
| Goat Anti-Mouse IgG H&L (HRP) | abcam | ab205719 |
| Anti-beta Actin antibody | abcam | ab8226 |
